# Gait Speed and Grip Strength Are Predictors of Cognitive Decline and Dementia in Older Individuals.

**DOI:** 10.1093/geroni/igab046.2460

**Published:** 2021-12-17

**Authors:** Suzanne Orchard, Joanne Ryan, Galina Polekhina, Elsdon Storey, Raj Shah, Trevor Chong, Anne Murray, Robyn Woods

**Affiliations:** 1 Monash University, Melbourne, Victoria, Australia; 3 Rush University Medical Center, Illinois, United States; 4 Hennepin HealthCare Research Institute, Minneapolis, Minnesota, United States

## Abstract

Lower gait speed and grip strength are common in older adults. However, the results of lower motor function on cognitive outcomes have been mixed. We examined the longitudinal association between baseline slow gait speed and weak grip strength, alone and in combination, with risk of incident dementia or cognitive decline in a cohort of older adults. Participants (n=19,114) aged 70 and over (65 if U.S. minority) without documented evidence of dementia, significant cognitive impairment, physical disability or previous cardiovascular disease at baseline, were recruited from community settings. Incident dementia was adjudicated by an expert panel using DSM-IV criteria. Incident cognitive decline was defined as a persistent intra-individual decline in score of >1.5 SD from baseline on any of the cognitive tests. Using cox proportional hazard models, slow gait speed at baseline was associated with an increased risk of dementia (63%) and cognitive decline (43%), over a median 4.7 years. Weak grip strength was not as strong a predictor, but was also associated with risk (43% and 11%, respectively). Both outcomes showed higher risk for dementia than cognitive decline. There was no gender-specific interaction. When considered together (adjusted for one another), gait speed and grip strength were both independently associated with cognitive decline and dementia. The synergistic association of these physical measures, each of which is readily administered in the clinic or home, serve as effective early markers of increasing risk of cognitive decline and incident dementia and thus, should be considered for routine health assessments for older adults.

